# On the need for accurate IUCN data and transparent methods: Response to Lin et al. (2025)

**DOI:** 10.1111/cobi.70380

**Published:** 2026-08-30

**Authors:** Monica V. Biondo, Ricardo Calado

**Affiliations:** ^1^ Fondation Franz Weber Bern Switzerland; ^2^ ECOMARE—Laboratory for Innovation and Sustainability of Marine Biological Resources, CESAM—Centre for Environmental and Marine Studies, Department of Biology University of Aveiro Aveiro Portugal

## INTRODUCTION

The article “Extent of Threats to Marine Fish from the Online Aquarium Trade in the United States” by Lin et al. (2025) addresses a relevant topic in the marine aquarium trade, a multimillion dollars industry that has in the European Union and the United States two of its main importing markets in terms of value (Biondo et al., 2024). We commend the authors for addressing the conservation implications of this trade, but we identified several factual inaccuracies in their reporting of species’ conservation statuses and data sources that may significantly affect the conclusions they drew.

## ERRORS IN IUCN RED LIST CLASSIFICATION

Lin et al. report that 45 marine fish species sold online are of conservation concern, including four species listed as endangered (*Pterapogon kauderni*, *Elacatinus prochilos*, *Gobiodon citrinus*, and *Amphiprion clarkii*), 16 as vulnerable, and 33 as having decreasing population trends. We found multiple inconsistencies between the conservation statuses listed by Lin et al. and those reported in the International Union for Conservation of Nature (IUCN) Red List (IUCN, 2025). The errors identified are not restricted to obscure, rarely traded species but actually apply to some iconic, routinely traded species (Biondo & Burki, 2019; Biondo et al., 2024). For example, *A. clarkii*, reported by Lin et al. as being endangered, was assessed globally and officially by the IUCN as of least concern. The IUCN reported it as being endangered only in the Persian Gulf. Similarly, *G. citrinus* was globally assessed as being of least concern and endangered only in the Persian Gulf. *Elacatinus prochilos* was globally assessed as being vulnerable and was considered endangered only in the Gulf of Mexico. Of the four reef fish species reported by Lin et al. as being endangered, only *P. kauderni* is indeed listed as globally endangered on the IUCN Red List. A complete list of these discrepancies as reported by Lin et al. and corrections based on the IUCN Red List (accessed 28 October 2025) are in Table 1.

**TABLE 1:** Species available for sale, their conservation status as provided by Lin et al. (2025), and corrections and additional information based on the International Union for Conservation of Nature (IUCN) Red List as of 28 October 2025.

		Lin et al.‐reported status	Corrections		
Species	Source		Global conservation status	Population status	Last global assessment
*Acanthurus chronixis* ^a^	Wild	Vulnerable	Vulnerable	Unknown	3 May 2010
*Acanthurus sohal* ^a^	Wild	Vulnerable	Least concern (vulnerable in the Persian Gulf, assessed 4 November 2013)	Stable	4 May 2010
*Zebrasoma xanthurum* ^a^	Wild	Vulnerable	Least concern (vulnerable in the Persian Gulf, assessed 3 November 2013)	Stable	7 May 2010
*Pterapogon kauderni* ^a^	Both	Endangered	Endangered	Decreasing	1 March 2007
*Ophioblennius steindachneri* ^a^	Wild	Vulnerable	Least concern	Stable	27 May 2007
*Coryphopterus personatus* ^a^	Wild	Vulnerable	Vulnerable	Unknown	22 March 2011
*Elacatinus figaro* ^a^	Cultured	Vulnerable	Vulnerable	Decreasing	6 April 2022
*Elacatinus prochilos* ^a^	Wild	Endangered	Vulnerable (endangered in the Gulf of Mexico, assessed 10 January 2014)	Unknown	01 March 2010
*Gobiodon citrinus* ^a^	Wild	Endangered	Least concern (endangered in the Persian Gulf, assessed 3 November 2013)	Unknown	28 June 2018
*Oxymonacanthus longirostris* ^a^	Wild	Vulnerable	Vulnerable	Decreasing	5 July 2015
*Lachnolaimus maximus* ^a^	Cultured	Vulnerable	Vulnerable	Decreasing	4 February 2009
*Coris aygula* ^a^	Wild	Vulnerable	Least concern	Unknown	12 April 2008
*Halichoeres marginatus* ^a^	Wild	Vulnerable	Least concern (vulnerable in Persian Gulf, assessed 5 November 2013)	Unknown	25 March 2009
*Holacanthus clarionensis* ^a^	Cultured	Vulnerable	Vulnerable	Stable (CITES App. II)	10 October 2009
*Amblyglyphidodon ternatensis* ^a^	Wild	Vulnerable	Vulnerable	Decreasing	15 Novembre 2010
*Amphiprion clarkii* ^a^	Both	Endangered	Least concern (endangered in the Persian Gulf, assessed 5 November 2013)	Unknown	03 February 2021
*Amphiprion mccullochi* ^a^	Cultured	Vulnerable	Vulnerable	Decreasing	1 August 2021
*Chrysiptera hemicyanea* ^a^	Both	Vulnerable	Vulnerable	Decreasing	1 August 2021
*Chrysiptera parasema* ^a^	Wild	Vulnerable	Least concern	Decreasing	23 September 2021
*Dascyllus trimaculatus* ^a^	Wild	Vulnerable	Least concern (vulnerable in the Persian Gulf, assessed 5 November 2013)	Unknown	23 September 2021

^a^ Names link to IUCN Red List entries.

Although *Conservation Biology* published a correction (14 November 2025), in this correction, Lin et al. primarily provide textual amendments and summary adjustments to the reported results, and several corrected values represent substantial quantitative changes rather than minor discrepancies. Notably, the number of vulnerable species was changed from 16 to 12, and the number of species not recognized as aquarium trade taxa by the IUCN or FishBase nearly tripled (increased from 100 to 282), altering both the severity and nature of the conservation risk narrative. Critically, the correction does not correct Appendix S8, which remains incomplete, listing only a subset of species and lacking accurate conservation status data. This issue is particularly important because many marine ornamental fishes are listed as either not evaluated or data deficient on the IUCN Red List or their assessments are over 10 years old, potentially limiting the reliability of conservation status data. As a result, Appendix S8 continues to hinder replication of analyses and verification of the main findings. Future work would benefit from clearer separation of global versus regional IUCN assessments and greater transparency in how conservation categories are harmonized across databases. Readers should refer to the corrected values in Table 1 when considering policy implications.

## METHODOLOGICAL TRANSPARENCY

In their Methods section, Lin et al. provide insufficient detail on how conservation statuses were assessed. In their Appendix S1, the authors say refer “extinction risk, population trend, and use/trade data were obtained from IUCN in November 2023 and updated in August 2025.” Given the errors reported above, our attempts to replicate Lin et al.’s methods and extract data from IUCN yielded very different conclusions about the global conservation status of the species Lin et al. refer to as imperiled (as shown in Table 1). Verification of IUCN Red List conservation status based on direct examination of information in the IUCN Red List (https://www.iucnredlist.org) would likely have prevented this type of mistake.

## PROBLEMS WITH DATA INTERPRETATION

Only 20 of the 45 species Lin et al. claimed to be of conservation concern are listed in their Appendix S8 (referred to as Appendix A8 in the text but labeled Appendix S8 in the appendix file). Of the 16 species they identified as vulnerable, seven are currently classified as least concern on the IUCN Red List. The claim that the United States accounts for two‐thirds of the global marine ornamental fish trade is not supported by the cited literature. Published global estimates range from 15 to 30 million fish annually (Biondo & Burki, 2020; Monticini, 2010; Wabnitz et al., 2003). The sources cited for the two‐thirds figure either offer no quantitative data (Sinha et al., 2023) or report U.S. imports of about 10 million fish in 2011 (Rhyne et al., 2012) and an average of 7.5 million for 2008, 2009, and 2011 (Rhyne et al., 2017). Although 10 million imports would represent two‐thirds of the lowest global estimate (15 million), this proportion falls to 33–50% within the broader 20–30 million range supported by most analyses. Thus, the assertion is inconsistent with available data. Additionally, Appendix A4 (S4 in the appendix file) includes no species names, even though 100 traded fish species are absent from both the IUCN Red List and FishBase aquarium trade records.

## IMPLICATIONS

Accurate representation of conservation statuses is critical in studies of wildlife trade. Misreporting IUCN categories can distort the perceived level of threat, potentially leading to misinformed conservation priorities, policy decisions, and public perception. A reanalysis of all species found in trade by Lin et al. based on verified IUCN Red List conservation status data is essential to ensure that Lin et al.’s conclusions regarding the marine aquarium trade are realistic and to ensure transparent and reproducible methods are used to address this important marine conservation topic.
